# Political Considerations When Monitoring the Commercial Determinants of Health: Comment on "National Public Health Surveillance of Corporations in Key Unhealthy Commodity Industries – A Scoping Review and Framework Synthesis"

**DOI:** 10.34172/ijhpm.8777

**Published:** 2024-12-18

**Authors:** Jennifer Lacy-Nichols, Alexandra Jones, Katherine Cullerton

**Affiliations:** ^1^Centre for Health Policy, Melbourne School of Population and Global Health, The University of Melbourne, Melbourne, VIC, Australia.; ^2^Faculty of Medicine, University of New South Wales, Sydney, NSW, Australia.; ^3^The George Institute for Global Health, Barangaroo, NSW, Australia.; ^4^School of Public Health, The University of Queensland, Brisbane, QLD, Australia.

**Keywords:** Corporate Political Activity, Monitoring, Transparency, Commercial Determinants, Political Feasibility

## Abstract

Better intelligence about how commercial actors influence health and equity is crucial to advance public health policies that prioritise people over profits. The "template surveillance system" proposed by Bennett and colleagues presents a useful step towards increasing our knowledge about the practices of commercial actors and monitoring potentially harmful activities. Yet practical considerations around what sectors or commercial practices are prioritised, how we gather this intelligence, and who "owns" or funds surveillance initiatives bring to the fore political challenges with this work. Here, we reflect on our own experience researching corporate political activities (such as lobbying) to present recommendations for advancing the "template surveillance system" proposed by Bennett and colleagues.

## Introduction

 We applaud the push from Bennett et al^1^ for advancing an agenda to monitor commercial practices. Their study developed a *“template surveillance system to be used by national governments across industries”* (p. 2). Below, we make proposals to move this agenda forward and flag practical difficulties encountered in our own experiences working in what Bennett et al label the “political environment.” In particular, we offer suggestions to look beyond the “harmful trinity” of unhealthy commodity industries and to monitor corporate political activities, while noting the potential conflicts of interest which may arise from the blurring of the commercial and political worlds, including challenges around relying on government funding in this field.

## Looking Beyond the “Harmful Trinity” of Tobacco, Alcohol, and Ultra-processed Foods

 Bennett and colleagues make it clear that they are interested in more than just the “unhealthy commodity industries” focused on in their scoping review. They observe that “the public health harms of commercial actors span to other industries, including pharmaceuticals, firearms, social media and financial institutions, and go beyond commodities to practices and use of power”^1^ (p. 2). The case for widening the gaze of commercial determinants of health (CDoH) scholarship is argued compellingly in the 2023 *Lancet* series on CDoH.^2,3^ While there is robust evidence of the harms arising from tobacco, alcohol, ultra-processed foods and fossil fuels,^2^ the next generation of CDoH scholarship must look beyond this narrow slice of the commercial world. Yet with a few exceptions, the academic community has lagged in turning its attention to new commercial sectors.^4^

 One of the arguments of *The Lancet* series on CDoH is that any company or industry sector will influence health outcomes, albeit in different ways.^3^ Box 1 illustrates three examples of health harms arising from diverse industry sectors. The intersectional, multi-industry approach advanced by Bennett et al is valuable as it can help to identify patterns and commonalities across industry sectors or types of commercial actors (such as industry associations or lobby groups). We have previously found it useful to differentiate between types of political activities (such as lobbying and political donations) and examine which actors do (or do not) engage in political activities. Surprisingly, we have sometimes found that the largest companies or industry associations do not engage, although this finding may also highlight a gap in transparency regulations that make political activities less visible or harder to track.^5^ For public health advocacy groups with limited resources, knowing which commercial actors are likely to oppose policies or engage in lobbying can help focus their own counter-advocacy.

**Box 1. Health Harms of Diverse Industries**

**Fashion:** Historical concerns about unsafe working conditions and human rights violations in the garment industry have become exacerbated by the rise of “fast fashion” (ie, cheap and readily available clothes) and its unrelenting workloads and deadlines.^6^ Fast fashion has likewise increased textile waste and placed further stress on agricultural lands (eg, water usage in cotton production and water contamination from chemicals).^6^ Beyond these supply chain issues, the fashion industry, through its marketing, is linked to body image issues, weight stigma, and unhealthy relationships with food.

**Data analytics:** A myriad of concerns have been raised about use and misuse of data ranging from social media companies designing algorithms to make their apps addictive to youth, to facilitating cyberbullying, hate speech and disinformation. Concerns about youth suicide have reached a level that the US Surgeon General proposed a warning label on social media akin to tobacco.^7^ Likewise, data privacy is a growing concern as companies have been criticised for selling consumer data. Particular concerns have been raised about health data (such as collected from Fitbits and other personal devices), and whether health insurance companies could use it to identify and discriminate against people with pre-existing health conditions.^8^

**Housing & construction:** Housing is both a human right and a multi-trillion-dollar asset. This tension between social good and wealth generation stymies efforts to improve living conditions for vulnerable groups. The private rental market raises issues of poor-quality housing, with one study observing “Providing slum housing for the poorest is a highly profitable activity but housing them in decent conditions [is] a real challenge.”^9^ Poorly regulated private construction firms operating in an environment of austerity (eg, lack of enforcement of building regulations) have led to health and safety concerns, vividly shown in the Grenfell fires in the United Kingdom.^10^ Internationally, construction workers (often migrants) may be subjected to violence, hazardous living conditions and have limited access to healthcare and education.

While any company or industry sector will influence health outcomes, given limited resources it will be important to prioritise monitoring efforts. Countries face different health burdens, and future CDoH scholarship and monitoring can begin to tease out how different commercial actors and industry sectors contribute to a range of health harms in a jurisdiction. Thinking of our own research on corporate political activities, in addition to being guided by a country’s health burden it will be useful to consider a country’s specific political and economic context. We can think of this as being led by power instead of health.

 For instance, it may be useful to consider which companies or sectors of the economy are especially important to a country’s gross domestic product, as that likely signals the potential influence those industries may have in politics. Australia, for instance, has large and lucrative mining and agricultural sectors that are heavily export oriented. While commercial entities involved here are ripe for analysis, there is a strong likelihood that governments will not be motivated to monitor and disclose the activities of industries that provide significant tax, royalty revenue and/or employment (not to mention political donations to political parties). On the other hand, scrutiny of financial, supply chain and employment practices may be critical to assess whether the company or industry’s narrative about jobs and economic growth is indeed as beneficial as claimed. Investigative journalists, for instance, have revealed the tax minimisation practices of many large companies, which suggest that the economic benefits that some companies claim may not pan out in practice (especially for low- and middle-income countries where many of their supply chains are located).^13^ This highlights an important opportunity for public health monitoring of commercial practices to hold governments accountable for ensuring that companies operating within their borders are not acting in ways that undermine health – or in the case of tax minimisation, undermine the economic prosperity of their people.

 Beyond government reticence, we note there is a real chance that monitoring and exposing the harms of powerful industries may risk the safety of researchers and activists. Research about the experience of tobacco control advocates in low- and middle-income countries found that most had experienced intimidation (such as legal threats) and more than 40% had experienced more aggressive forms of intimidation such as theft, physical intimidation and cyber-attacks.^12^ While these experiences are perhaps uncommon as a general rule in CDoH research, they draw attention to the need for institutional protection of researchers and activists, especially those working on more controversial topics such as weapons.

## Monitoring Political Practices – Recommendations From Our Experience

 In the case of monitoring political practices (Bennett and colleagues’ “political environment”), much of the necessary data originates from democratic accountability and transparency mechanisms. These include things like political donation reporting, lobbyist registers, publication of submissions to public consultations, and open access to diaries of government representatives. These mechanisms, and supporting tools such as freedom of information legislation, are important to promoting public trust in government, and ensuring that decision-making is fair, honest, and free of undue interference, including from corporations. In the absence of accessible, timely, comprehensive and high-quality data, monitoring commercial political practices is a non-starter.

 In many parts of the world, including developed democracies such as Australia, these mechanisms are still suboptimal despite longstanding advocacy to improve them.^14^ Fulfilment of Bennett and colleagues’ vision of public health surveillance will require acceleration of reform in this sphere, which sits beyond the realm of traditional public health advocacy. Those interested in the CDoH can coordinate with other strategic civil society actors (eg, environmental, human rights or transparency activists for example) who may have been more traditionally active in this space. Beyond general democratic reforms, CDoH advocates may be inspired by legislative initiatives such as updated mandated corporate sustainability reporting requirements in the European Union which from 2024 require large and listed companies to publish regular reports on how their activities impact people and the environment, including the effects of the corporation on human health.^14^ While national reform is valuable, as Bennett et al acknowledge, global collaboration to create international standards, data collection processes, and networks will enable more comprehensive monitoring.

 In parallel, researchers can explore existing underutilised data sources or emerging techniques to pilot surveillance methods and support the call for improved data. Existing underutilised data sources include business and financial datasets (such as the Panama and Paradise Papers, Orbis, Compustat, and Pitchbook). Emerging tools include automated methods of web scraping, natural language processing to analyse large datasets (eg, policy submissions) and the largely untapped potential of generative AI (eg, to map political networks). Especially for work on the revolving door (the movement between public and private sector employment), new and creative methods to advance scholarship and regulation are needed. This is an area where we are especially interested to explore new methods and datasets, including tools to make datasets available and interactive, such as Power BI—a business intelligence tool that provides visual tools to monitor data patterns and track key metrics. In our previous work we have piloted Power BI and Google Data Analytics to move away from static charts towards something more akin to weather or fitness dashboards.^5^

 This is also where we can learn from the non-governmental organisation (NGO) sector. Bennett and colleagues’ scoping review looked only at the academic literature, when many of the richest and longest-lasting examples of monitoring come from NGOs and civil society. While Bennett et al note some examples (eg, the US Right to Know’s investigation of industry influence on research and “Revolving Door Watch” by Corporate Observatory Europe), future studies could systematically analyse these approaches for learnings. In our own work, we are exploring NGO and media strategies to measure and share data on the revolving door for a range of industry sectors, expanding the toolset for corporate political activity research. There are terrific and long-standing initiatives (such as Tobacco Tactics, Corporate Accountability, ACT Promoção da Saúde, and Southeast Asia Tobacco Control Alliance) that have pioneered innovative approaches to monitoring the practices of particular industries that could have broader applicability.

## Political Considerations When Deciding Which Commercial Practices to Prioritise and Who Should “Own” the Monitoring Initiative

 One of the more interesting findings is the unevenness with which different commercial practices were or were not included in frameworks (Figure 2 of Bennett et al). Thirteen of 14 frameworks included corporate social responsibility, while only one included tied development aid. While this shows the uneven attention in the academic literature to commercial practices, just because some practices have historically been sidelined does not necessarily mean they are less deserving of scrutiny.

 Criteria to assist with prioritisation may include size of impact on health, scope or prevalence of the corporation and/or practice within the country, feasibility of monitoring given available resources/data, and the potential to take policy action in response to any resulting findings. This is acknowledged in the paper as a next step.

 We argue that one key factor that should be considered when prioritising practices to monitor is feasibility. As noted above, one element of this is technical feasibility ie, the practical availability of data by which to conduct surveillance. Technical feasibility may also consider whether there are potential implementable and effective solutions available to address any resulting findings, and the related costs of developing and implementing these.^15^ Feasibility may be demonstrated by implementation of best-practice regulations elsewhere. In the case of corporate lobbying for example, Canada provides an example with robust cooling off rules that limit government officials from moving directly into lobbying roles when they leave government.

 Considerations of feasibility also encompass political feasibility, which is more difficult to assess. Public policy scholar Charles Lindblom was a proponent of incrementalism and suggested that the feasibility of a new policy solution to be accepted relates strongly to current policy.^15^ The more radical a departure from the current negotiated position, the harder it will be to sell. With this in mind, the proposal from Bennett et al seems incredibly unfeasible given the current inaction already noted above from most governments around the world regarding the implementation of disclosure and transparency measures on political donations and lobbying, for example. This inaction persists despite many years of advocacy from academics and NGOs for the adoption of such measures. Nonetheless, in the case of political transparency there are policy champions, like Canada, Ireland, Scotland, and Chile that provide examples of where more robust transparency measures have been implemented and could be progressively strengthened. Two of the authors are part of an ongoing project to benchmark government transparency of lobbying with the aim of developing practical recommendations to strengthen government disclosure mechanisms to make political activities more visible (a first step towards regulating them).

 One recommendation for advancing the template surveillance system from a political feasibility perspective is to engage with those in government bureaucracies or administrations, such as policy officers, but also politicians, to assess feasibility. Bennett et al foresee this in their proposal for a Delphi study. If the answer is “no,” it may assist to determine if some aspects of their proposed system are more politically acceptable than others. As per Lindblom’s incrementalist approach, we could focus on implementing one aspect at a time. If feasibility is demonstrated, this may then lead to the adoption of other aspects of the monitoring system.

 A final consideration is who should “own” the monitoring system. Bennett et al argue that while the monitoring of health impacts of corporations has, to date, mainly been the tasks of civil society and academia, governments should take an increased role as part of their responsibility for public health monitoring in all other domains. They also note that it is governments who hold unique authority to shape the regulatory environment in which corporations operate.

 We agree in principle that governments should, at minimum, resource the monitoring of CDoH. However, we note that some aspects of CDoH research and monitoring—those concerning commercial practices in the political environment in particular—may shine a light on undue commercial influence on governments. Again, the revolving door is a vivid example of the blurring between commercial and political worlds.

 Just as there has been considerable scholarship highlighting the risks/limitations of industry self-regulation, we note that governments may be unwilling to shine a light on themselves where potentially corrupt or conflicted activities may exist. For this reason, we suggest that government funding to extend current civil society efforts may be a useful middle ground that expands monitoring activities without the potential conflicts of interest that can arise where governments monitor themselves. In parallel, we suggest that governments require more comprehensive and timely data disclosure to support greater transparency (both by themselves and commercial actors). By investing in independent monitoring of commercial practices, this may foster greater trust in the surveillance system.

## Concluding Thoughts

 We (along with many of our colleagues around the world) agree that commercial actors and their practices require far more attention, scrutiny and ultimately robust regulation than the current status quo provides. We also note that many aspects of preventing and controlling non-communicable diseases are political. The CDoH are overtly so. Commercial actors pay taxes (read, government revenue), employ citizens (voters) and deliver public services (in a privatised economy). Beyond this, many are wealthy and well-connected politically. The determinants of health—social, economic, political, and commercial—are fundamentally interconnected. Potential conflicts of interest must be carefully considered – not only from commercial actors, but from within government (often due to their close relationships with commercial actors).This is necessary for a nuanced and thoughtful approach to monitoring the CDoH. We hope that our reflections on the challenges and opportunities for public health surveillance of CDoH provide a useful resource for future efforts.

## Ethical issues

 Not applicable.

## Conflicts of interest

 JLN is the recipient of a fellowship from the Victorian Health Promotion Foundation.
